# First-in-human SPECT/CT imaging of [^211^At]PSMA-5: targeted alpha therapy in a patient with refractory prostate cancer

**DOI:** 10.1007/s00259-024-07017-w

**Published:** 2024-12-17

**Authors:** Tadashi Watabe, Koji Hatano, Sadahiro Naka, Hidetaka Sasaki, Takashi Kamiya, Yoshifumi Shirakami, Atsushi Toyoshima, Jens Cardinale, Frederik L. Giesel, Kayako Isohashi, Norio Nonomura, Noriyuki Tomiyama

**Affiliations:** 1 Department of Radiology, Graduate School of Medicine, Osaka University, Suita, Japan; 2 Institute for Radiation Sciences, Osaka University, Suita, Japan; 3 Department of Urology, Graduate School of Medicine, Osaka University, Suita, Japan; 4 Department of Pharmacy, Osaka University Hospital, Suita, Japan; 5 Department of Radiology, Osaka University Hospital, Suita, Japan; 6 Department of Nuclear Medicine, University Hospital Dusseldorf, Dusseldorf, Germany

**Figure:**
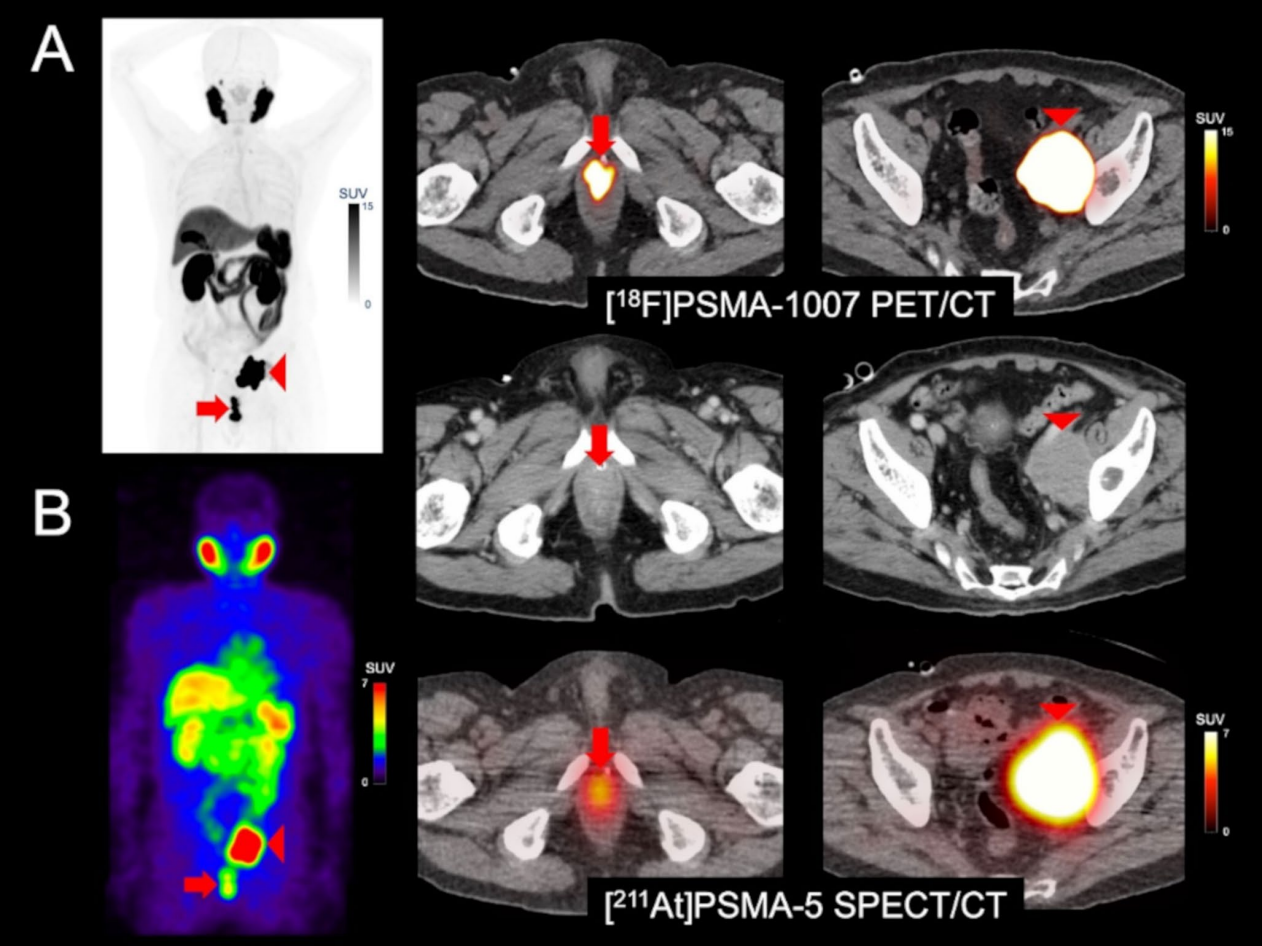

Astatine (^211^At) is an alpha emitter with a physical half-life of 7.2 h and can be produced using a 30 MeV cyclotron [1]. The global supply infrastructure for astatine is expanding, aiming to establish a reliable supply chain that supports routine clinical use. We developed and completed a preclinical evaluation of [^211^At]PSMA-5 [1] and have successfully initiated an investigator-initiated Phase 1 clinical trial (NCT06441994).

Here, we report the first-in-human SPECT/CT image of [^211^At]PSMA-5 in a patient. [^211^At]PSMA-5 was administered to a man in his 70s with metastatic castration-resistant prostate cancer refractory to standard treatment including androgen receptor signaling inhibitors, docetaxel, and cabazitaxel. SPECT/CT imaging was performed 3 h post-administration using a VERITON-CT (Spectrum Dynamics Medical) equipped with a full-ring cadmium zinc telluride (CZT) detector, targeting the 79 keV X-rays from the daughter nuclide of ^211^Po.

Pre-treatment [^18^F]PSMA-1007 PET/CT (A) and [^211^At]PSMA-5 SPECT/CT (B) images showed similar distribution patterns, with high uptake in recurrent/metastatic lesions (left: maximum intensity projection, right: fusion and contrast-enhanced CT images). Both images revealed high accumulation in the soft tissue mass within the prostate area (SUVmax = 60.7 on [^18^F]PSMA-1007 PET and 4.9 on [^211^At]PSMA-5 SPECT) (arrows) and in the enlarged left external iliac lymph node metastasis (SUVmax = 143.7 and 17.6, respectively) (arrow heads). Physiological accumulation was similarly observed in both modalities in the salivary glands, liver, spleen, small intestine, and kidneys, with no detectable urinary excretion. This image provides proof-of-concept for a theranostic approach using the ^18^F/^211^At-labeled compound pair.

## Data Availability

The original data presented in this study are available and further inquiries can be directed to the corresponding author.
